# Dynamic Properties of Microresonators with the Bionic Structure of Tympanic Membrane

**DOI:** 10.3390/s20236958

**Published:** 2020-12-05

**Authors:** Yongpeng Tai, Kai Zhou, Ning Chen

**Affiliations:** 1 College of Automobile and Traffic Engineering, Nanjing Forestry University, Nanjing 210037, China; 2 College of Mechanical and Electronic Engineering, Nanjing Forestry University, Nanjing 210037, China; zhoukai@nse.cn.com (K.Z.); chenning@njfu.com.cn (N.C.)

**Keywords:** dynamic properties, microresonator, bionic structure, tympanic membrane

## Abstract

The structure of a microresonator will affect the vibration characteristics and the performance of the system. Inspired by the structural characteristics of the human tympanic membrane, this paper proposed a microresonator with the bionic structure of a tympanic membrane. The structure of a tympanic membrane was simplified to a regular shape with three structural parameters: diameter, height, and thickness. To imitate the tympanic membrane, the contour surface of the bionic structure was modeled based on the formula of transverse vibration mode of a circular thin plate. The geometric model of the bionic structure was established by using the three structural parameters and the contour surface equation. The dynamic properties of the bionic model were studied by the finite element method (FEM). We discuss the modal characteristics of the bionic structure and study the effect of structural parameters and scale on the dynamic properties. The advantages of the bionic structure were investigated by a comparison with circular plate microresonators. The results illustrate that the bionic structure can significantly improve the resonant frequency and have a much larger effective area of vibration displacement.

## 1. Introduction

A microresonator is a typical high-precision MEMS (microelectromechanical system) component, which has excellent mechanical resonance characteristics and stable performance. It is widely used in aerospace, precision measurement, national defense and military, communication, and other fields [1,2,3,4,5]. Microresonators play an important role in high-frequency oscillators, filters, and sensors (accelerometer, gyroscope, etc.). Common microresonators are microbeams [3,6], microrings [7], and microplates [8], which are relatively simple and regular in terms of geometric structures. With the development of MEMS, microresonators with complex three-dimensional structures have been designed to meet the performance requirements such as microhemisphere [9], shallow arch microbeam [10], slot structure [11], laminated structure [12], and so on. The shape and structure characteristics of these microresonators can affect their dynamic performance and determine the advantages and disadvantages in design and application.

The main design parameters of a microresonator are resonance frequency, quality factor (*Q*), and energy conversion efficiency. The resonance frequency is determined by many factors such as geometry, material properties, prestress, surface roughness, etc. For very high-frequency systems, the resonator with large mass and low elastic coefficient is no longer applicable, therefore, it is necessary to design resonant devices with reasonable geometry and resonant modes. The quality factor of microresonators is mainly related to three dissipation mechanisms: air damping, support loss, and thermoelastic damping. When the air pressure is larger than 1 mTorr, the air damping becomes of great significance [13]. In general, air damping and support loss can be ignored by vacuum packaging and optimization design. However, thermoelastic damping is the intrinsic damping of materials and cannot be eliminated, but it is related to the geometry and vibration mode. The capacitive energy conversion method is commonly used for MEMS resonators. In order to increase the energy conversion efficiency, the area of the capacitor plate can be increased or the distance between the plates can be reduced. Accordingly, for high-frequency and high-performance microresonators, the influence of geometric structure on resonant frequency, energy dissipation, and energy conversion efficiency should be considered in the design.

In recent years, the combination of MEMS and bionics has become a new research direction including the design and manufacture, miniaturization, and intellectualization of bionic MEMS devices. Many MEMS sensors are designed by imitating biological structures, for example, bionic MEMS acoustic positioning sensors, velocity and direction sensors, tactile sensors, displacement sensors, accelerometers, vector underwater acoustic sensors, and so on [14,15,16,17,18,19,20]. The combination of MEMS and bionics has solved many technical problems and created many new MEMS devices.

For some vibration sensing apparatus in organisms, the geometric structure is gradually optimized in the evolution process, which has excellent dynamic properties. It is worthy to research bionic resonators based on these biological structures. The tympanic membrane is an important component of the auditory apparatus of humans and other quadrupeds, which is a complex biomechanical system with the function of sound reception and perception [21,22,23]. Usually, the sound energy is received and converted into the mechanical vibration of the ossicular chain by the tympanic membrane and then transmitted to the inner ear to cause hearing. From the perspective of shape, the tympanic membrane is a thin, cone-shaped membrane, which is different from some common resonators. This paper imitated the special contour of a tympanic membrane, and proposed a microresonator with the bionic structure of a tympanic membrane. The structural parameters were obtained by simplifying the geometry of the tympanic membrane, and the contour surface equation was fitted by the Bessel function. We hope that the bionic structure can be an alternative to the plate resonator in some MEMS applications and show good performance. Although the bionic structure is much more complex than common microresonators that are fabricated by planar technologies, it is similar to the microhemisphere resonator as a shell of revolution. Therefore, the three-dimensional fabrication process [2,24] can be used to fabricate the bionic structure.

The rest of this paper is organized as follows. Section 2 proposes the bionic structure model based on the tympanic membrane and gives an expression of the contour surface by using the Bessel function. Section 3 studies the vibration modal of the bionic structure for the case of fully clamped boundary conditions. Section 4 discusses the effect of geometry and scale on the dynamic properties of the bionic structure and compares the bionic structure with a circular plane. The last section contains a few concluding remarks of this work.

## 2. Bionic Structure Model

### 2.1. Structural Parameters

**Figure 1:**
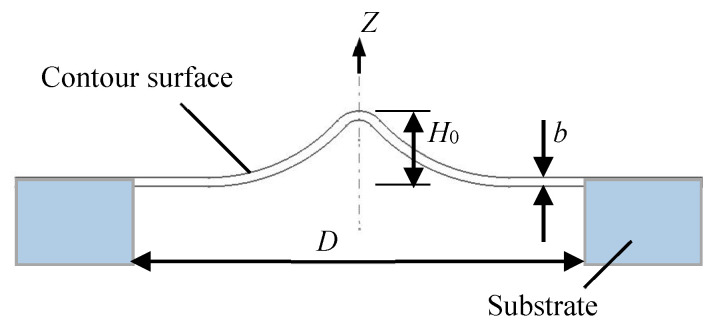
Schematic diagram of the microresonator with the bionic structure of the tympanic membrane.

The shape of the tympanic membrane is irregular and uneven, which connects with other tissues in the human ear. The tympanic membrane can be treated as a conical shape with a diameter, height, and thickness. According to CT (computed tomography) imaging of the human tympanic membrane [25], the diameter of the tympanic membrane varies from 9.01 mm to 9.76 mm, the height gradually increases from the edge to the center with the maximum of 2.42 mm, and the thickness is not consistent, ranging from 50 μm to 100 μm [26]. In order to facilitate the bionic structure design, we simplified the tympanic membrane structure to make it a thin-walled geometric structure with regular shape and uniform thickness. First, the surface of tympanic membrane is regarded as a flat, uniform, and smooth surface whereas the contour shape remains. Second, the edge of tympanic membrane is regarded as a regular circle. Then, the bionic structure model of the tympanic membrane is obtained as shown in Figure 1. According to the bionic model, there are three main structural parameters: diameter *D*, height *H*_0_, and thickness *b*. The proportion of the tympanic membrane structure can be preserved by using the three parameters. In this paper, we selected a set of typical values within a reasonable range to investigate the dynamic properties of the bionic structure of *D* = 9.38 mm, *H*_0_ = 2.42 mm, and *b* = 50 μm.

As the MEMS device is at the micron scale, the size of the bionic structure model of tympanic membrane is still too large. Therefore, we reduced the typical value by 50 times and obtained the structural parameters of the bionic model, as shown in Table 1.

### 2.2. The Contour Surface Equation

The key point of modeling a reasonable bionic structure of the tympanic membrane is to design a contour surface by using the structural parameters. The bionic structure of the tympanic membrane can be regarded as a thin-walled rotating body, and its contour surface is very similar to the first-order transverse vibration mode of a circular plane constrained by fully clamped boundary conditions. Thus, we can fit the contour surface of the tympanic membrane structure by using the vibration mode.

To obtain the contour surface equation, we first considered the transverse vibration of a circular thin plate with fixed boundary conditions. According to the theory of vibration mechanics, the formula of the transverse vibration mode of circular thin plate with radius *R* is [27]

(1) $$H(r)=C_{1}J_{n}(\beta r)+C_{2}I_{n}(\beta r)$$

where *r* is the distance from the center of the circular plate; *β* is the coefficient related to the natural frequency; *C*_1_ and *C*_2_ are undetermined coefficients; and *J*_0_(*βr*) and *I*_0_(*βr*) are 0-order Bessel function of the first kind and the modified Bessel function of the first kind, respectively, given by

(2) $$J_{0}(\beta r)=\sum_{m=0}^{\infty }\frac{{\left(-1\right)}^{m}}{m!\Gamma (m+1)}{\left(\frac{\beta r}{2}\right)}^{2m}$$

(3) $$I_{0}(\beta r)=i^{-0}J_{0}(i\beta r)$$

For fully clamped boundary conditions, we have

(4) $$H\left(R\right)=0,{\frac{dH}{dr}|}_{r=R}=0$$

where *R* = *D*/2. Theoretically, the first mode of the transverse vibration is without nodal diameter and nodal circle, expressed as [27]

(5) $$H(r)=C_{1}J_{0}(3.196\frac{r}{R})+C_{2}I_{0}(3.196\frac{r}{R})$$

Since *C*_1_ is proportional to *C*_2_, the above equation can be expressed as

(6) $$H\left(r\right)=C[J_{0}(3.196\frac{r}{R})+BI_{0}(3.196\frac{r}{R})]$$

where *B* = −*J*_0_(3.196)/*I*_0_(3.196) ≈ 0.0557, and *C* is the undetermined coefficient.

Next, we designed the contour surface of the bionic structure based on Equation (6). According to the mode expression, the maximum displacement occurred at *r* = 0, then

(7) $$H\left(0\right)=C[J_{0}(0)+BI_{0}(0)]=H_{0}$$

Using the property of the Bessel function, we obtained *J*_0_(0) = *I*_0_(0) = 1. Thus, the parameter *C* can be expressed as

(8) $$C=\frac{H_{0}}{1+B}$$

Finally, the expression of the contour surface of the bionic structure was obtained, given by

(9) $$H\left(r\right)=\frac{H_{0}}{1+B}[J_{0}(3.196\frac{r}{R})+BI_{0}(3.196\frac{r}{R})]$$

**Figure 2:**
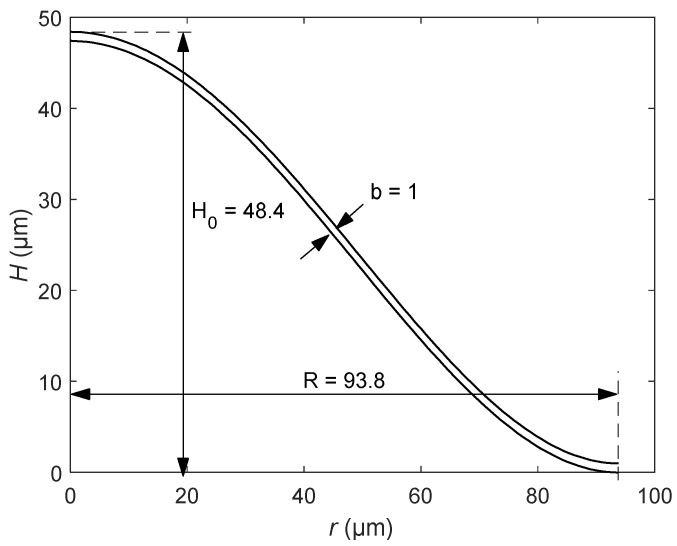
The contour curve of the bionic structure.

**Figure 3:**
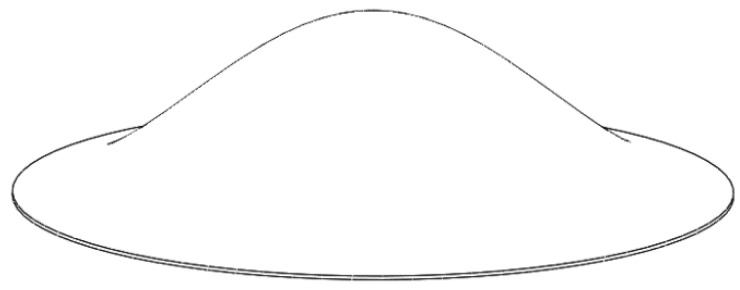
The three-dimensional model of the bionic structure of the tympanic membrane.

Note that only *R* and *H*_0_ are needed to establish the contour curve of the surface of the bionic structure. Substituting the structural parameters in Table 1 into Equation (9), the contour curve of a bionic structure can be obtained. Furthermore, we plotted the profile shape of the surface by considering the thickness, as shown in Figure 2. By rotating the curve around the *Z*-axis to produce a completely curved surface, the microresonator model of the bionic structure of the tympanic membrane was established, as shown in Figure 3.

Equation (9) illustrates that the contour curve of the bionic structure can be fitted by the Bessel function. Using the combination of the Bessel function of the first kind and the modified Bessel function of the first kind, Equation (9) can be extended to a general expression, which can describe more complex contour curves, given by

(10) $$H=\sum_{i}a_{i}J_{i}\left(\beta r\right)+\sum_{j}b_{j}I_{j}\left(\beta r\right)\;i\;,\;j\;=\;0,\;1,\;2\ldots$$

where *a_i_* and *b_j_* are variable parameters.

## 3. Modal Analysis

### 3.1. The FEM Model

**Figure 4:**
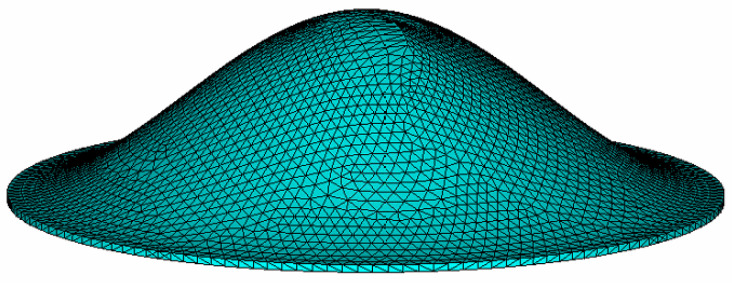
The finite element model of the bionic structure of the tympanic membrane.

Due to the complex geometry of the bionic structure, we established the finite element model of the bionic structure to analyze its vibration characteristics, as shown in Figure 4. The FEM simulation of this modal analysis as well as the harmonic analysis in the next section was solved by ANSYS, in which SOLID226 was used and hence, the thermoelastic damping was taken into consideration. In order to ensure the calculation accuracy, the mesh size of the finite element model was equal to the thickness of the bionic structure. Although there are three kinds of boundary conditions for the bionic structure model (i.e., fully clamped, simply supported and free boundary), we selected fully clamped boundary conditions in this paper. This is because, in some studies of the tympanic membrane, the periphery of the tympanic membrane is assumed to be fully clamped to the ear canal [21]. Since silicon is the main material of microresonators, we assumed that the bionic models in this paper were all made of Si material, and the material parameters are shown in Table 2.

### 3.2. Vibration Modes

**Figure 5:**
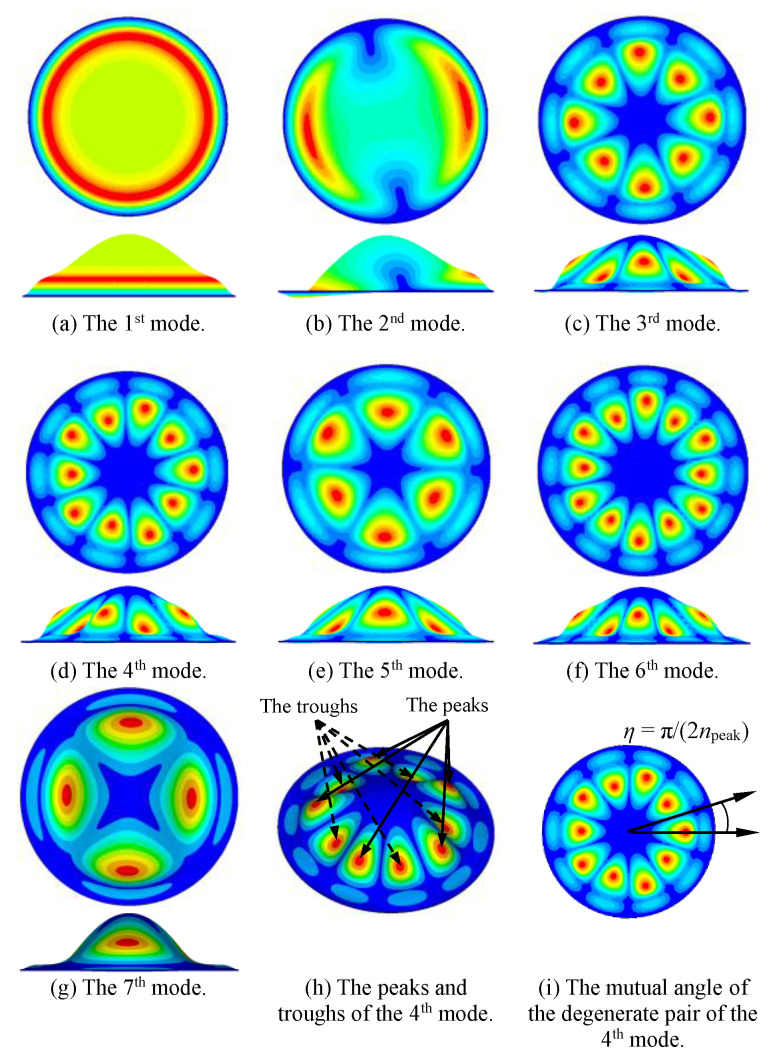
The modes of the bionic structure of tympanic membrane. (**a**–**g**) The first seven mode shapes. (**h**) The peaks and troughs of the 4th mode. (**i**) The mutual angle between the two degenerate resonance modes.

Using the finite element model, we calculated the first seven vibration modes of the bionic structure of the tympanic membrane. Figure 5 shows the diagrams of the vibration modes, and the corresponding natural frequency of each mode is listed in Table 3. 

From Figure 5a–g, the natural frequency increased and the vibration mode became more complex as the mode order increased. In the first mode, the maximum displacement was distributed continuously in a circular ring shape, whereas at the higher order mode, the maximum displacement was of scattered distribution in a circular ring. For all modes, the vibration displacement of the center part was small, especially for the case of higher-order modes. Note that the maximum displacement occurred on the side of the bionic structure where the real tympanic membrane connects the auditory ossicles and transmits mechanical vibration. We compared the vibration mode of the bionic structure to that of the real tympanic membrane [29,30]. It was found that the positions of the maximum displacements were very similar; however, because the structure of the tympanic membrane was not completely symmetrical and influenced by the ossicular chain, the overall vibration mode was quite different from that of the bionic structure. As the first-order mode is easy to excite and can store more mechanical energy, it is preferred for resonators. We focused only on the first-order mode in this study. 

For non-fundamental modes, mode shapes had certain regularity. Figure 5h shows that except for the fundamental mode, the peaks and troughs appeared in pairs. If there is a peak, there must be a trough. From Figure 5a–g, note that the mode number does not monotonically change with the peak number. For example, the 3rd mode had four peaks while the 7th mode had two peaks. We also found that due to the symmetric shell, an equivalent degenerate pair of modes with a mutual angle existed for each natural frequency except for the fundamental mode. Figure 5i shows the mutual angle *η* of the two degenerate modes of the 4th natural frequency. The angel *η* = π/(2*n*_peak_) is related to the peak number *n*_peak_ of each mode. As other axisymmetric MEMS resonators (microrings and microhemisphere), the mechanical energy of bionic structure in high-order modes can transfer between two degenerate resonance modes under the effect of Coriolis force. Therefore, the bionic structure is expected to be applied in gyroscope sensors based on the dynamic properties. Furthermore, the bionic structure is a three-dimensional shell with vibration displacement distributed in different planes, and hence has potential application in multi-axis microsensors. 

Next, we studied the direction of vibration at the maximum displacement of the first-order mode, which was a continuous ring and close to the edge of the bionic structure. Take an arbitrary point *M* on the ring of maximum displacement as an example, which is located at (*X*_1_, *Y*_1_, *Z*_1_) = (75.046, 0, 5.278). For point *M*, the relative displacement (*USUM*) and the components in three coordinate directions (*UX*_1_, *UY*_1_ and *UZ*_1_) can be calculated using the FEM simulation. Then, we obtain three angles *α*_1_, *β*_1_, and *γ*_1_, which represent the angles between the vibration direction of the *M* and *X*-axis, *Y*-axis, and *Z*-axis, respectively, and given by

(11) $$\alpha_{1}=\arccos\frac{UX_{1}}{USUM}=76.101^{{}^{\circ}}$$

(12) $$\beta_{1}=\arccos\frac{UY_{1}}{USUM}=90.01^{{}^{\circ}}$$

(13) $$\gamma_{1}=\arccos\frac{UZ_{1}}{USUM}=13.898^{{}^{\circ}}$$

According to Equation (9), the contour curve of the XOZ plane can be expressed as

(14) $$H\left(X\right)=\frac{H_{0}}{1+B}[J_{0}(3.196\frac{X}{R})+BI_{0}(3.196\frac{X}{R})]$$

The derivative equation of *H*(*X*) is

(15) $$H^{\prime }\left(X\right)=\frac{3.196}{R}\cdot \frac{H_{0}}{1+B}[-J_{1}(3.196\frac{X}{R})+BI_{1}(3.196\frac{X}{R})]$$

Since *M* is a point in the XOZ plane, *H*’(*X*_1_) = −0.5231 and the slope of the normal at this point is −1/*H*’ = 1.91168, which is also the tangent of the angle between the normal and the *X*-axis. Therefore, the angle between the normal and the *X*-axis is

(16) $$\theta_{1}=\arctan(-\frac{1}{H^{\prime }})=62.386^{{}^{\circ}}$$

As can be seen from Equations (11) and (16), *α*_1_ > *θ*_1_ and *α*_1_ – *θ*_1_ = 13.715°. Note that there is a difference between the vibration direction and the normal direction for the maximum displacement. This implies that the vibration mode of the bionic structure is not a pure transverse vibration, but a complex mode coupling with transverse and contour vibrations, which is determined by the curved surface of the bionic structure of the tympanic membrane. This results in a higher natural frequency than plan resonators with pure transverse vibration or a larger vibration displacement than some resonators with pure contour vibration. Furthermore, compared to transverse vibration, other components of the coupling mode can reduce thermoelastic damping caused by pure transverse vibration and improve the quality factor and sensitivity of the structure.

## 4. Results

For the bionic microresonator, the three main geometric parameters, *H_0_*, *b*, and *R* (*D*/2) can significantly affect dynamic properties. This section first studies the effect of three structural parameters and scales on the dynamic properties of bionic structures and then compares the dynamic properties of bionic structures with those of circular plates. The advantages of bionic structures of tympanic membranes are discussed. The material properties of bionic devices are shown in Table 2 and the surface load *P* = 10^−6^ sin*ωt* MPa was uniformly applied to the surface of the bionic structure. The force *P* is an empirical value that gives the vibration displacement within a reasonable range.

### 4.1. Effect of Height

The most significant difference between the bionic structure of the tympanic membrane and the circular plate is that the contour surface is curved with a maximum height at the center. Therefore, the variation of the maximum height *H*_0_ has a great effect on the performance of the bionic structure.

First, we considered the effect of different *H*_0_ on the natural frequency of bionic structures. Assume that the structural parameters are *R* = 93.8 μm, *b* = 1 μm, and *H*_0_ = 48.4 μm, 40 μm, 30 μm, 20 μm, 10 μm, and 5 μm. The first natural frequencies corresponding to different maximum heights *H*_0_ are 4.4901 MHz, 4.1671 MHz, 3.7019 MHz, 3.0620 MHz, 2.1805 MHz, and 1.5308 MHz, respectively. Note that the first natural frequency is proportional to the height *H*_0_.

**Figure 6:**
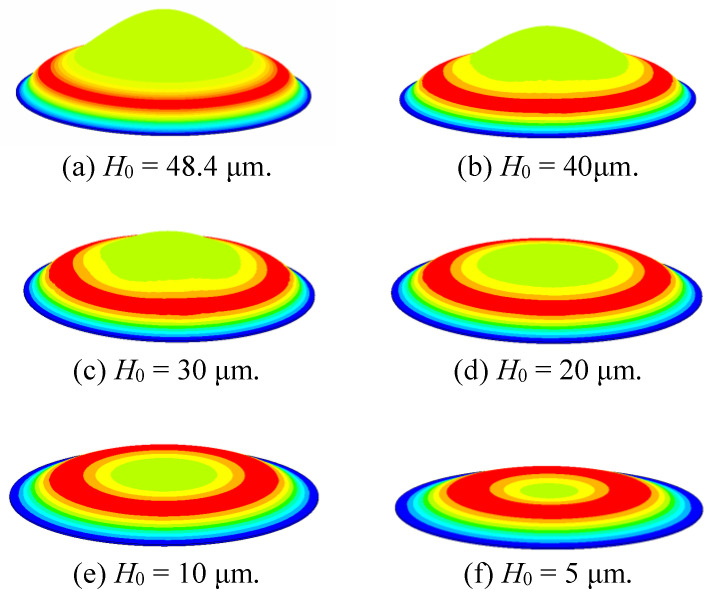
The mode shapes of the bionic structure for different values of *H*_0_.

Next, we studied the response of different *H*_0_ of bionic structures excited by the same harmonic force *P* at the first natural frequencies. Figure 6 shows the FEM simulation of vibration displacement at the first natural frequency for different *H*_0_. For all cases, the radius and thickness were constant. As shown in the figure, the amplitude distribution of the entire device was in the shape of a circular ring, and the amplitude was related to the distance from the central axis. The figure also shows that when *H*_0_ > 0, the maximum amplitude is a circular ring. As *H*_0_ decreases, the maximum amplitude moves toward the central axis. Note that even for very small *H*_0_ (5 μm), the maximum amplitude still exhibited an obvious ring shape. For the case of *H*_0_ = 0, the bionic structure degenerated into a circular plate, and hence the maximum amplitude became a point on the central axis.

**Figure 7:**
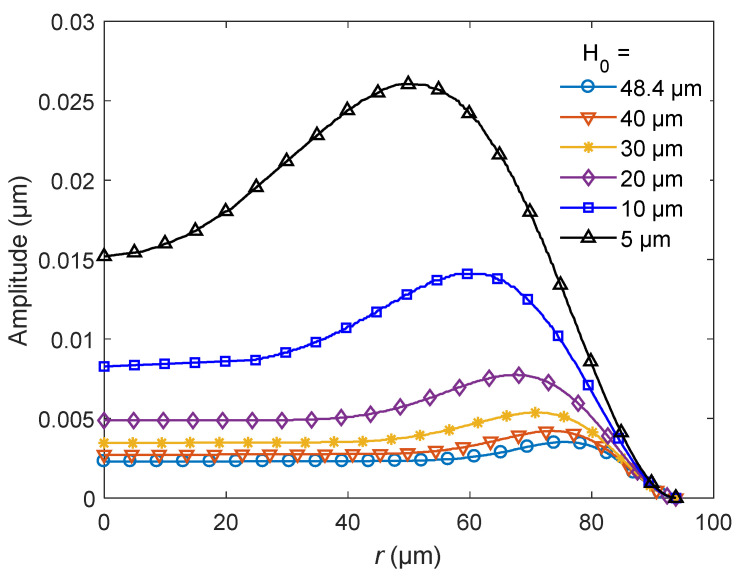
The amplitude distribution of the bionic structure for different heights H_0_.

Figure 7 shows the distribution of the vibration amplitude excited by the first natural frequency for different *H*_0_. As shown in the figure, the amplitude decreases with the increase of *H*_0_, and the position of the maximum amplitude is further away from the central axis with the increase of *H*_0_. Table 4 lists the relative distance of the maximum amplitude *r*_max_/*R* for different *H*_0_, where *r*_max_ is the distance between the maximum amplitude and the central axis. As shown in Table 4, when *H*_0_ approaches 0, *r*_max_/*R* tends to 0.

### 4.2. Effect of Thickness

Thickness is also a key factor affecting the dynamic performance of the bionic microresonator. We studied the effect of the thickness on the vibration characteristics of the bionic structure for constant *H*_0_ = 48.4 μm and *R* = 93.8 μm. First, we calculated the natural frequencies of the bionic structures with different thicknesses by FEM simulation. It was found that when the thickness *b* = 1 μm, 1.5 μm, 2 μm, 2.5 μm, and 3 μm, the corresponding first natural frequencies were 4.4901 MHz, 5.3975 MHz, 6.1305 MHz, 6.7480 MHz, and 7.2843 MHz, respectively. Therefore, the first natural frequency is proportional to the thickness *b*. 

**Figure 8:**
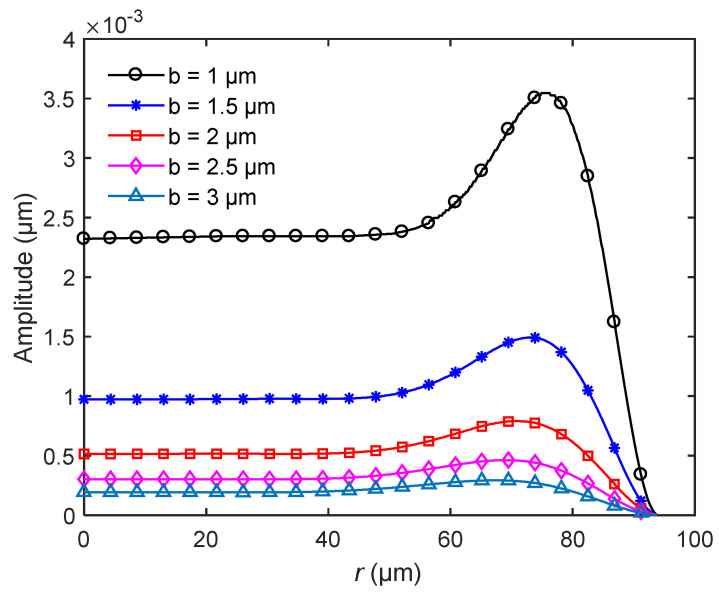
The amplitude distribution of the bionic structure for different thicknesses *b*.

Figure 8 shows the amplitude distribution of bionic structures with different thicknesses at the first natural frequencies. As shown in the figure, as the thickness of the bionic structure increases, the amplitude of the entire structure decreases. This is because the stiffness of the structure increases with the increase in the thickness. The figure also shows that the effect of the thickness on the relative position of the maximum amplitude is very weak. However, the thickness can significantly affect the value of the amplitude.

### 4.3. Effect of Radius

In this section, we consider the effect of radius *R* on the dynamic properties when the height is *H*_0_ = 48.4 μm and the thickness *b* = 1 μm. The first natural frequencies can be calculated by FEM simulation for different radii *R*. When *R* = 80 μm, 93.8 μm, 120 μm, 150 μm, and 200 μm, the corresponding first-order natural frequencies are 6.0003 MHz, 4.4901 MHz, 2.8041 MHz, 1.8419 MHz, and 1.0464 MHz, respectively. The larger the radius, the smaller the first natural frequency. 

**Figure 9:**
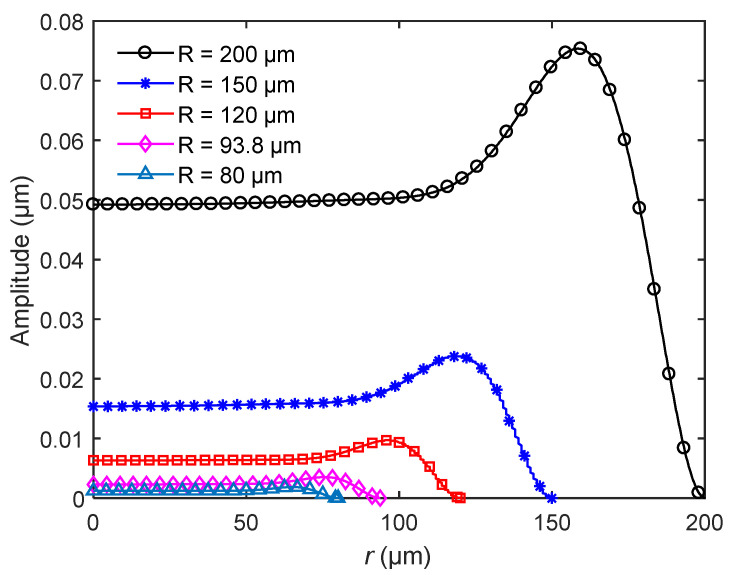
The amplitude distribution of the bionic structure for different radii *R*.

Figure 9 shows the amplitude distribution of bionic structures at the first natural frequencies under the same surface load *P*. From the figure, the radius can affect the amplitude significantly. The larger the radius, the greater the amplitude caused by vibration. In addition, with the change of radius, the maximum amplitude position of the bionic structure also changes. Table 5 lists the relative positions of the maximum amplitude of bionic structures with different radii. As shown from the table, although the radii are different, the relative position of the maximum amplitude is about 0.81 for each model, namely, the amplitude distribution of vibration is almost unchanged. 

### 4.4. Effect of Scale

**Figure 10:**
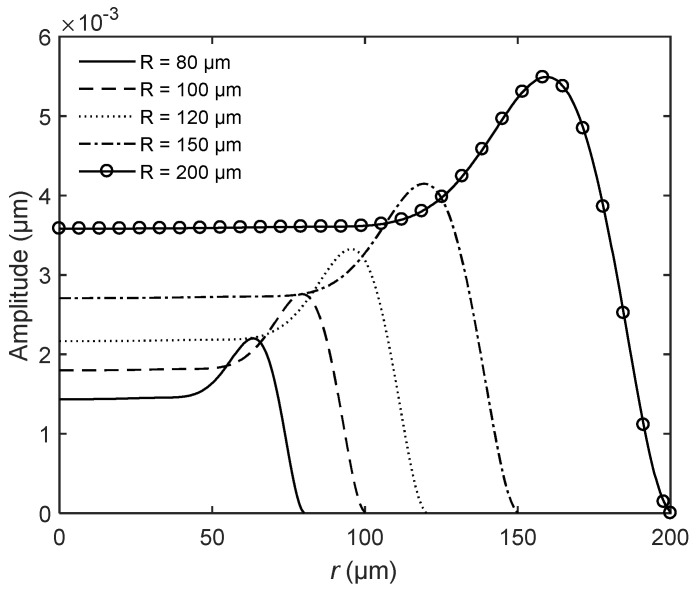
The amplitude distribution of the bionic structure for different scales with constant ratio *R*:*H*_0_:*b* = 80:40:1.

Next, we studied the scale effect on the dynamic properties of bionic microresonators. For MEMS devices, the scale effect will be remarkable and result in different characteristics. We kept the ratio between structural parameters constant at *R*:*H*_0_:*b* = 80:40:1 and changed the scale of the bionic structure by varying the radius *R* = 80 μm, 100 μm, 120 μm, 150 μm, and 200 μm. Under such conditions, the shape was completely consistent for different scales. The relationship between the scales and the first natural frequency is listed in Table 6. From the table, the first natural frequency decreases with the increase of scale. Figure 10 shows the amplitude distribution of different scales of bionic structure under the excitation *P* at the first natural frequency of each scale. As shown in the figure, the amplitude increases with the increase of the scale. Table 7 lists the relative magnitude of the maximum amplitude and the relative distance *r*_max_/*R* for different scales. This implies that the relative distance *r*_max_/*R* is constant for different scales and the maximum amplitude is proportional to the scale of the bionic structure. 

According to the above study, it was found that to design the bionic structure, we should tune three structure parameters: *H*_0_ (height), *b* (thickness), and *R* (radius), as these three parameters have a great influence on the amplitude of frequency. In the design, we first must consider the thickness limitation in the fabrication process, then the radius and height should be determined according to the required frequency and amplitude.

### 4.5. Comparison with Circular Microplane Resonator

In this section, we compare the bionic microresonator of the tympanic membrane with a circular microplate resonator and discuss the difference of vibration characteristics between the two devices under the same radius and the same first natural frequency. In fact, the bionic structure of the tympanic membrane can become a circular plate for the case of *H*_0_ = 0. Hence, the circular microplate can be regarded as a special case of the bionic structure in this paper.

**Figure 11:**
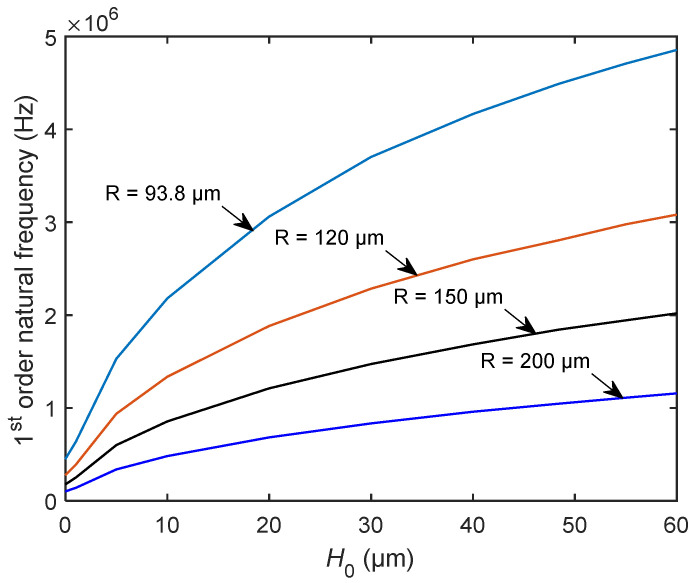
The variation of the first natural frequency of the bionic structure with the height *H*_0_ for different *R*.

We first compared the two kinds of resonators with the same radius. Figure 11 shows the relationship between the first natural frequency and the height *H*_0_. The specific parameters were radius *R* = 93.8 μm, 120 μm, 150 μm, and 200 μm and thickness *b* = 1 μm. As shown in Figure 11, when the radius is constant, the height *H*_0_ is directly proportional to the natural frequency of the bionic structure of the tympanic membrane. Therefore, when *H*_0_ gradually decreases, the natural frequency of the bionic structure also decreases. When *H*_0_ = 0, the bionic structure of the tympanic membrane becomes a circular plate, and its frequency is also reduced to the minimum. The figure also shows that the first natural frequency of the bionic structure decreases with the increase in radius when the height *H*_0_ is constant.

**Figure 12:**
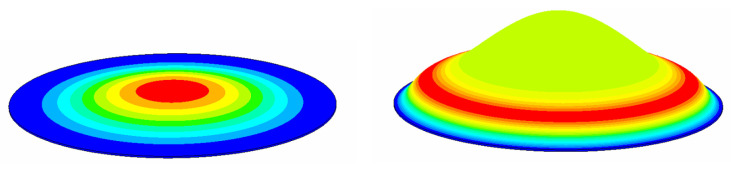
The mode shapes of the circular plate and the bionic structure. (**a**) The circular plate. (**b**) The bionic structure.

Figure 12 shows the first-order mode shapes of the bionic structure and the circular plate with the same radius. As shown in the figure, the displacement of the circular plate is mainly concentrated in the center, whereas for the bionic structure of the tympanic membrane, the amplitude distribution is circular around the center of the structure. Note that the maximum amplitude of the circular plate is a single point, but that of the bionic structure is a complete circular ring formed by points. Therefore, compared with the circular plate, the range of maximum amplitude of bionic structure is larger.

Next, we compared the natural frequencies of the bionic structure and circular plate for the cases of different radii and boundary conditions. In the application of microresonators in engineering, in addition to the fixed edge constraints, there are also simply supported constraints. Table 8 lists the first natural frequencies of circular plates and bionic structures. It can be seen from the table that the natural frequency of the circular plate with a simple support was about 1/2 of that of fixed constraint, whereas that of the bionic structure was about 1/3 of that of the fixed constraint. The natural frequency of the bionic structure was about five to seven times higher than that of the circular plate. This is because the circular plate is pure transverse vibration, whereas the tympanic membrane bionic structure is the coupling of transverse and contour vibration. The increase in radius *R* can significantly reduce the natural frequencies of the two devices but has little effect on the frequency ratio of the two devices.

**Figure 13:**
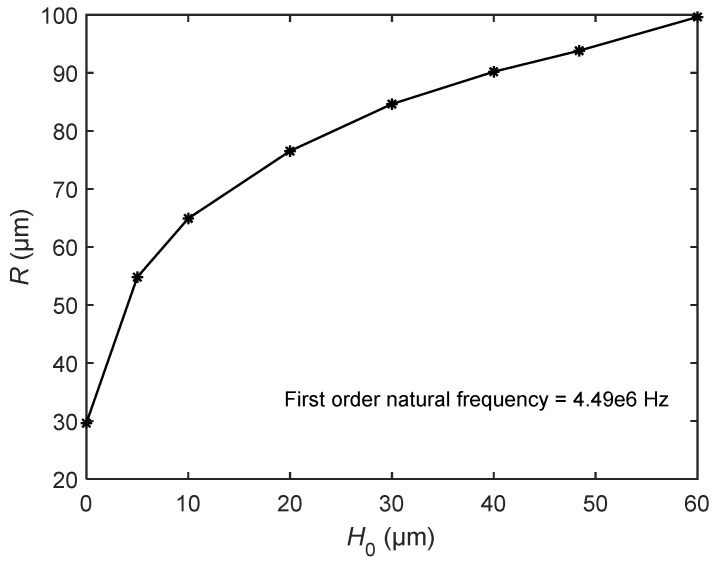
The relationship between radius *R* and height *H*_0_ when the first natural frequency is constant.

Next, we considered the case that the bionic structure and the circular plate had the same first natural frequency. The parameters of the bionic structure were *R* = 93.8 μm, *H*_0_ = 48.4 μm, *b* = 1 μm, and the first natural frequency was 4.49 × 10^6^ Hz. Figure 13 shows the relationship between radius and height when the first natural frequency is constant. Therefore, we obtained the structure parameters of the circular plate as *R* = 29.63 μm, *H*_0_ = 0, and *b* = 1 μm.

**Figure 14:**
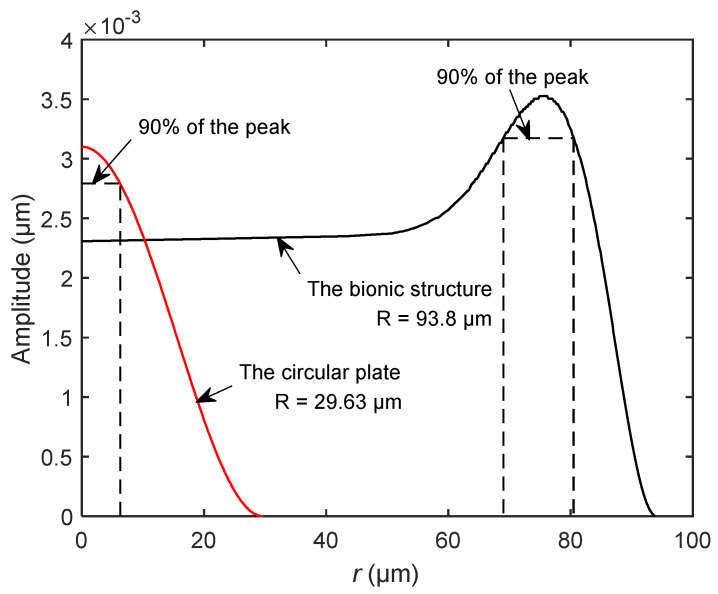
The comparison of vibration amplitudes between the circular plate and the bionic structure for the case of the same natural frequency.

Figure 14 shows the amplitude distribution curves of the bionic structure and the circular plate under the same natural frequency and the same surface load *P*. As shown in the figure, the maximum amplitude of the bionic structure is a little larger than that of the circular plate. To estimate the energy conversion efficiency, we assumed that the effective vibration displacement is greater than or equal to 90% of the maximum amplitude, which can provide a strong enough signal for the detection element. For the circular plate, the projected area of the effective vibration displacement on the horizontal plane is 36π μm^2^, which accountes for 4.1% of the projection area of the structure. For the bionic structure, the projected area of the effective vibration displacement is 1937π μm^2^, accounting for 22.02% of the total projected area. In this case, the effective vibration displacement of the bionic structure is about five times larger than that of the circular plate. The larger area leads to lower driving voltage and higher sensitivity, and the sensitive electrode can easier capture the measurement signal. This implies that the bionic structure can obtain a larger range of effective vibration than the circular plate. Accordingly, the bionic structure of the tympanic membrane is expected to be more efficient for energy conversion due to its unique contour surface and vibration mode, which can bring the advantages of excitation and measurement in practical application.

## 5. Conclusions

In this paper, a new kind of microresonator with a bionic structure of tympanic membrane was proposed. We simplified the real regular structure of the tympanic membrane properly, summed up the necessary structural parameters, and fitted the contour curve with the Bessel function. In order to study the dynamic characteristics, the dynamic model of the bionic structure was established by using FEM. The modal analysis showed that the vibration mode is a coupling with transverse and contour vibrations. The effects of height, thickness, radius, and scale on the vibration characteristics were investigated. Compared with the circular plate, it was found that the natural frequency of the bionic structure was about five to seven times higher. Moreover, under the same natural frequency, the proportion of effective vibration displacement of the bionic structure was larger, and the energy conversion efficiency was higher in theory. Due to the dynamic properties of the bionic structure, this type of microresonator is expected to be applied as actuators, gyroscope sensors, and so on. 

## Figures and Tables

**Table 1:** The structural parameters of the bionic model of the tympanic membrane.

Diameter *D* (μm)	Height *H*_0_ (μm)	Thickness *b* (μm)
187.6	48.4	1

**Table 2:** The material properties of polysilicon at 300 K [28].

Parameters	Polysilicon
Young’s modulus, *E* (GPa)	157
Poisson’s ratio, *υ*	0.22
Density, *ρ* (kg m^−3^)	2330
Thermal conductivity, *κ* (W m^−1^ K^−1^)	90
Specific heat, *C_p_* (J kg^−1^ K^−1^)	699
Thermal expansion coefficient, *α* (K^−1^)	2.6 × 10^−6^

**Table 3:** The first seven natural frequencies of the bionic model.

Mode	The Natural Frequencies (MHz)
1	4.4901
2	5.0863
3	5.7668
4	6.1232
5	6.4361
6	7.1902
7	8.1132

**Table 4:** The relative distance of the maximum amplitude for different heights *H*_0_.

Parameters	Values						
*H*_0_ (μm)	48.4	40	30	20	10	5	0
*r*_max_/*R*	0.8054	0.7826	0.7538	0.7202	0.6534	0.5532	0

**Table 5:** The relative distance of the maximum amplitude for different radii *R*.

Parameters	Values				
*R* (μm)	80	93.8	120	150	200
*r*_max_/*R*	0.8151	0.8101	0.8099	0.8133	0.7923

**Table 6:** The first natural frequencies of the bionic structures of different scales with constant ratio *R*:*H*_0_:*b* = 80:40:1.

No.	*R* (μm)	*H*_0_ (μm)	*b* (μm)	The First Natural Frequencies (MHz)
1	80	40	1	5.6183
2	100	50	1.25	4.4718
3	120	60	1.5	3.7176
4	150	75	1.875	2.9683
5	200	100	2.5	2.2234

**Table 7:** The maximum amplitude of bionic structures for different scales.

No.	*R* (μm)	Scale	*r*_max_/*R*	Relative Maximum Amplitude
1	80	1	0.792	1
2	100	1.25	0.793	1.254
3	120	1.5	0.795	1.511
4	150	1.875	0.795	1.886
5	200	2.5	0.798	2.497

**Table 8:** Comparison of the first natural frequencies between the circular plate and the bionic structure.

Boundary Conditions	*R* (μm)	The First Natural Frequencies (MHz)		Ratio
		Circular Plate	Bionic Structure	
Fully clamped	100	0.7912	5.5358	0.1429
	150	0.3516	2.5964	0.1354
	200	0.1977	1.4903	0.1326
Simple supported	100	0.3722	1.8895	0.1970
	150	0.1654	8.8700	0.1865
	200	0.9306	5.0977	0.1825
